# Validation of verbal autopsy methods for assessment of child mortality in sub-Saharan Africa and the policy implication: a rapid review

**DOI:** 10.11604/pamj.2019.33.318.16405

**Published:** 2019-08-22

**Authors:** Chigozie Jesse Uneke, Henry Chukwuemeka Uro-Chukwu, Onyedikachi Echefu Chukwu

**Affiliations:** 1 African Institute for Health Policy and Health Systems, Ebonyi State University, PMB 053 Abakaliki, Nigeria

**Keywords:** Verbal autopsy, validation, child mortality, sub-Saharan Africa

## Abstract

Reliable data on the cause of child death is the cornerstone for evidence-informed health policy making towards improving child health outcomes. Unfortunately, accurate data on cause of death is essentially lacking in most countries of sub-Saharan Africa due to the widespread absence of functional Civil Registration and Vital Statistics (CRVS) systems. To address this problem, verbal autopsy (VA) has gained prominence as a strategy for obtaining Cause of Death (COD) information in populations where CRVS are absent. This study reviewed publications that investigated the validation of VA methods for assessment of COD. A MEDLINE PubMed search was undertaken in June 2018 for studies published in English that investigated the validation of VA methods in sub-Saharan Africa from 1990-2018. Of the 17 studies identified, 9 fulfilled the study inclusion criteria from which additional five relevant studies were found by reviewing their references. The result showed that Physician-Certified Verbal Autopsy (PCVA) was the most widely used VA method. Validation studies comparing PCVA to hospital records, expert algorithm and InterVA demonstrated mixed and highly varied outcomes. The accuracy and reliability of the VA methods depended on level of healthcare the respondents have access to and the knowledge of the physicians on the local disease aetiology and epidemiology. As the countries in sub-Saharan Africa continue to battle with dysfunctional CRVS system, VA will remain the only viable option for the supply of child mortality data necessary for policy making.

## Introduction

Within the past few decades there has been a substantial progress in the reduction of child mortality globally. A 2017 report from the United Nations Inter-agency Group for Child Mortality Estimation (IGCME) indicated that, global under-five mortality rate dropped to 41 deaths per 1,000 live births in 2016 from 93 in 1990, which is a notable 56% decline [1]. However, disparities still exist in the global trend of child mortality reduction across different regions of the world. Recent statistics from the World Health Organization (WHO) Global Health Observatory Data Repository showed that in sub-Saharan Africa, infant, neonatal and under-five mortality rates per 1000 live births reduced from 93.5, 40.9 and 154 in 2000 to 53.9, 27.7 and 79.5 in 2015 respectively [2]. Despite these appreciable reductions, the child mortality rate in sub-Saharan Africa remains unacceptably high. The sub-region, is still the home to all six countries (Central African Republic, Chad, Mali, Nigeria, Sierra Leone and Somalia) with an under-five mortality rate above 100 deaths per 1,000 live births [1, 2]. Furthermore, the probability that a child, aged 5 dies before reaching his or her fifteenth birthday in sub-Saharan Africa is 17 times higher than the average in high-income countries [1].

Clearly, sub-Sharan Africa requires accelerated progress if the region will achieve the Sustainable Development Goal (SDG) targets in child survival. According to WHO, reliable data on the levels and causes of child mortality are cornerstones for building a solid evidence base for health policy, planning, implementation, budgeting, resource allocation, monitoring and evaluation, which are core prerequisites to improving child health outcomes [3]. Unfortunately, accurate, reliable and timely available data on Cause of Death (COD) among children is essentially lacking in most of sub-Saharan Africa. One major reason for this drawback in the sub region as in other Low and Middle-Income Countries (LMICs) is the widespread near absence of well-established and functional Civil Registration and Vital Statistics (CRVS) systems [4]. All across the sub-Saharan African countries especially in the rural and underserved areas and even in urban areas where the CRVS systems do not function, many of the child deaths and the COD are not recorded. This is largely because the majority of deaths occur at home and there is little chance that deaths occurring away from health facilities will be recorded and the cause of death certified [3, 5]. As a partial solution to this problem, Verbal Autopsy (VA) has gained global prominence as a primary strategy for obtaining information about COD in populations where CRVS and medical certification are absent [3, 6-10]. The WHO defined VA as a method used to determine the COD based on an interview with next of kin or other caregivers of the deceased [3]. According to Desai and colleagues [7], VA tools typically consist of a structured survey administered to a close relative or associate of the deceased by a trained field worker, to record the signs and symptoms that occurred before death and this information is used to assign the most probable COD, most often via Physician-Certified Verbal Autopsy (PCVA) coding.

The WHO is promoting the application of VA especially in regions such as the sub-Saharan Africa where the CRVS systems and medical certification are weak [2, 10]. This is because the increased use of VA for routine application in national health information systems has been reported by Murray and co-workers [6], to have the potential to greatly improve the availability of reliable and essential information on causes of death for disease control programs. The WHO noted in a recent publication on VA standards, that there has been a proliferation of interest, research and development in all aspects of the VA process, including VA data-collection systems, VA questionnaires content and format, cause of death assignment process, coding and tabulation of causes of death, and validation of VA instruments [3]. Consequently, there exist various methods of VA application [11] and many more methods are being developed. Some of the methods which have been reported in literature include: the PCVA [12], artificial neural networks model [13], data-derived algorithms [14], probabilistic approach [15], InterVA model [16], symptom pattern method [17], King Lu method [18], robust metrics model [19], cause-specific mortality fractions model [20], tariff method [21] and random forests model [22]. Despite the value of VA as a potential alternative for COD data for child mortality for resource constrained setting like the sub-Saharan Africa, there are a number of fundamental issues associated with its accuracy, reliability and routine applicability.

First, Murray and colleagues argued that there are widespread concerns about VA in terms of the dependability of symptom information collected from families and the practicality of relying on physicians to review anonymous symptom-based questionnaires [6]. They further noted that confidence in VA as a legitimate data collection mechanism is limited because it is not known how accurately the method can diagnose the underlying cause of death compared with hospital-based procedures or how different approaches to VA perform in assigning causes of death [6]. Second, Menendez and co-workers noted that verbal autopsies have been questioned because they are subject to a high degree of misclassification errors, especially for conditions with poor diagnostic specificity such as most causes of peri-neonatal deaths [23]. Third, Byass and colleagues observed that there has been substantial methodological heterogeneity involved in VA, which can magnify existing uncertainties over cause-specific mortality [24]. Fourth, Leitao and co-workers noted that the expanding use of VA in generating mortality data has led to a proliferation of different VA instruments (comprising a set of questions/indicators that elicit pertinent information on signs, symptoms and circumstances preceding death and a corresponding list of COD that has impaired data comparability across sites and over time [5]. Another major challenge with the use of VA in resource scarce setting has to do with concerns about its validity. VA validation has been defined as a process that compares an Underlying Cause of Death (UCOD) derived from the VA with a reference UCOD for the same death whereby the reference UCOD can be derived from pathological autopsy which is considered as a “gold standard”, or from clinical records which are considered as the next best alternative [25, 26]. Due to the importance of the validation of VA, a number of studies have been conducted in sub-Saharan Africa on the subject with variations in the findings of the investigations. Thomas and colleagues [4] advised that the findings from such studies need careful consideration, while Tran and co-workers [25] noted that establishing the validity of VA methods is important when considering to use these methods on a routine basis to improve the availability and quality of mortality data in the country. The objective of this paper is to review studies that investigated the validation of verbal autopsy methods for assessment of child mortality in sub-Saharan Africa with the view to providing scientific evidence relevant to policy making as it affects COD and child survival in sub-Saharan Africa.

## Methods

We conducted a MEDLINE PubMed search of literature in June 2018 and sought studies published in English that investigated the validation of VA methods in sub-Saharan Africa within the last three decades (1990-2018). We used the following keywords for the search: “verbal autopsy”, validation, child, mortality, Africa; these resulted to 17 publications. The identified publications were subjected to study inclusion criteria which included: (i) study must be conducted in any of the countries in sub-Saharan Africa; (ii) the study must not be earlier than 1990; (iii) the study must be based on the validation of one or more methods of VA; (iv) the study must be focused on children, infants and newborn; (v) the study must be a primary study either prospective or retrospective. One of the authors (CJU) reviewed the 17 publications identified and extracted data and other relevant information from those that fulfilled the study inclusion criteria. The other two authors (HCU and OEC) subsequently performed independent review of the identified publications and selected those that are in line with the study inclusion criteria from which data was extracted. Thereafter all the authors discussed and harmonized selected publications and arrived at a consensus regarding included studies. A total of nine out of the 17 identified publications fulfilled the study criteria and were included in the review. The reference sections of all the included studies were reviewed for any additional relevant study and other information. This process yielded additional five studies which satisfied the study inclusion criteria and making the included study a total of 14. The included publications were thereafter categorized into three groups (1990-1999; 2000-2009; 20^10^-2018) in Table 1, Table 2 and Table 3 according to the following information: author/year of publication; country; scope of validation; study design; target population; evidence generated; and policy implication.

**Table 1:** Profile of scientific publications in 2010-2018 that investigated verbal autopsy validation in sub-Saharan Africa

Author/year/reference	Country	Target population	Study design	Scope of validation	Evidence generated	Policy implication
Adewemimo *et al.* 2017 [27]	Nigeria	Newborn (1-27 days), children 1-59 months and under five	Prospective	Assessment of level of agreement between the EAVA and PCVA. All-cause mortality rates calculated from NDHS by applying VASA mortality fractions to NDHS all-cause rates.	EAVA and PCVA are very useful in providing direct estimates of the causes and determinants of neonatal and 1–59month mortality.	Data generated by EAVA and PCVA can serve to help guide the improvement of maternal, neonatal and child health policies and intervention programs
Mpimbaza *et al.* 2015 [28]	Uganda	Newborn (1-27 days) and children 1-59 months	Prospective	Evaluation of intra-rater reliability of PCVA. Also compared the accuracy of PCVA, Non-hierarchical and hierarchical algorithm in relation to the hospital diagnosis	High intra-rater reliability (83% agreement). PCVA had high specificity for neonatal (>67%) and child death (>83%). Hierarchical was better than the non-hierarchical algorithm	PCVA remains a reliable method for determining cause of death, with high repeatability.
Kalter *et al.* 2015 [29]	Niger	Newborn (1-27 days) and children 1-59 months	Retrospective	Compared PCVA with expert algorithm	Both had similar neonatal death cause distribution with Sepsis, Birth asphyxia, Pneumonia ranked highest. Cause distribution for child death differ between both methods.	Reasonable agreement was found between expert algorithm and PCVA.
Ndila *et al.* 2014 [30]	Kenya	Children 28 days to 14 years	Retrospective	PCVA and InterVA-4 was validated against diagnosis.	6% & 5% of deaths were attributed to SCDusing PCVA and InterVA respectively. Agreement coefficient for SCD between PCVA and hospital diagnosis was 95.5% while that for InterVAis 96.9%	VA may be useful in quantifying the contribution of SCD to childhood mortality in rural African communities.
Mpimbaza *et al.* 2011 [31]	Uganda	Newborn (1-27 days) and children 1-59 months	Prospective	Compared PCVA with hospital diagnosis	Sensitivity of VA in determining malaria death was 61% in Tororo and 50% in Kampala, its specificity was >88% in both locations. Its PPV varied widely between the two locations; 83% and 34% respectively.	VA provides acceptable level of accuracy for determining malaria deathat the population level in the setting of high and medium transmission.
Oti *et al.* 2010 [16]	Kenya	Children <5years	Retrospective	Compared InterVA and PCVA	The level of agreement between individual causes of death assigned by both methods was only 35% (kappa = 0.27, 95% CI: 0.25 - 0.30).	InterVA model showed promising results as a community-level tool for generating cause of death data from VAs

PCVA= physician certified verbal autopsy; VASA= verbal/social autopsy; VA= verbal autopsy, EAVA= expert algorithms verbal autopsy

SCD= sickle cell disease; InterVA= computer models to facilitate interpreting verbal autopsy; NDHS= national demographic health survey

**Table 2:** Profile of scientific publications in 2000-2009 that investigated verbal autopsy validation in sub-Saharan Africa

Author/year/reference	Country	Target population	Study design	Scope of validation	Evidence generated	Policy implication
Edmond *et al.* 2008 [32]	Ghana	Newborn (1-27 days)	Prospective	Compared PCVA with hospital diagnosis	VA performed poorly in stillbirth diagnosis. For neonatal deaths, its sensitivity was >60% for major causes of death while its specificity is 76% for Birth asphyxia but >85% for prematurity and infection.	Diagnostic accuracy of VA tool should be assessed in other regions and in multicenter studies.
Setel *et al.* 2006 [33]	Tanzania	Perinatal, newborn and under-5 children	Prospective	Compared PCVA with hospital diagnosis	VA underestimated stillbirth and overestimated malaria death in post newborn. No other significant differences between hospital diagnosis and VA.	VA reliably estimated CSMF for diseases of public health importance.
Fantahun *et al.* 2006 [34]	Ethiopia	Infants <1year and children 1-14years	Prospective	Compared result of InterVA with PCVA	Both showed prematurity/low birth weight, perinatal causes and pneumonia/sepsis as the major cause of death in infants; whereas, for the older children are pneumonia/sepsis, diarrheal diseases/malnutrition and malaria.	Compared with the PCVA, InterVA model is much less tedious and yet offers 100% consistency.
Kahn *et al.* 2000 [35]	South Africa	Under-5 children and Adults >15years	Retrospective	Compared PCVA with hospital diagnosis	No significant difference seen between them. For communicable diseases, VA had sensitivity, specificity and PPV of 69%, 96% and 90% respectively whereas for non-communicable diseases the values were 75%, 91% and 86% respectively	Evidence showed that VA is reliable. However, needs to be validated.

PCVA= physician certified verbal autopsy; VA= verbal autopsy; CSMFs=cause-specific mortality fractions; InterVA= computer models to facilitate interpreting verbal autopsy

CSMF= cause specific mortality fraction; PPV= positive predictive value

**Table 3:** Profile of scientific publications in 1990-1999 that investigated verbal autopsy validation in sub-Saharan Africa

Author/year/reference	Country	Target population	Study design	Scope of validation	Evidence generated	Policy implication
Quigley *et al.* 1996 [36]	Kenya	U5 children	prospective	Cause of death assessed by expert and data-derived algorithms and both were compared with hospital diagnosis	Data-derived diagnostic algorithm can give comparable or better diagnostic accuracy than expert algorithm. However, both gave high specificity.	Logistics regression and other methods for deriving algorithm should be explored in a wider setting.
Mobley *et al.* 1996 [37]	Namibia	U5 children	Retrospective	Compared VA diagnosis to hospital diagnosis	The sensitivities and specificities for the 5 causes of death studied were: malnutrition 73% and 76%, cerebral malaria 72% and 85%, malaria 45% and 87%, diarrhea 89% and 61%, Cough with dyspnea 72% and 64%, measles 71% and 85%.	VA can be useful in ascertaining the leading cause of death in childhood.
Nykanen *et al.* 1995 [38]	Malawi	Children	Retrospective	Compared VA diagnosis to hospital diagnosis	High sensitivity and specificity for the most common cause of childhood death.	VA is a promising method for collecting population-based information about child mortality
Todd *et al.* 1994 [39]	Gambia	Children	Retrospective	VAs were reassessed by the same Physicians and diagnosis compared. Also VA diagnosis was compared to hospital diagnosis	In 38 out of 141 VAs assessed the first and subsequent diagnosis differed, 44 out 94 diagnosis matched with the hospital diagnosis.	Poor sensitivity and specificity of VA here is due to the effect of malaria which presents like other causes.
Snow *et al.* 1992 [40]	Kenya	Children	Prospective	Compared PCVA diagnosis to hospital diagnosis	VA detected the common cause of death with specificities >80%. Its sensitivity for measles, neonatal tetanus, malnutrition trauma-related deaths was >75% but <50% for malaria, anemia, acute respiratory tract infection, gastroenteritis and meningitis.	VA used in malaria-specific interventions should be interpreted with caution.

PCVA= physician certified verbal autopsy; VA= verbal autopsy

## Current status of knowledge

### Physician-certified verbal autopsy most widely used verbal autopsy method

The result of this review clearly showed that PCVA is the most widely used VA method to determine child mortality COD in sub-Saharan Africa. This explains why all the studies [27-35, 36-40] with the exception of one [36] focused on the validation of the PCVA. Although PCVA has proven to be a very useful strategy to determine COD for children, infants and neonates in many parts of sub-Saharan Africa where there is no other better alternative, the limitations of the tool are well-established. Among the major drawbacks of the PCVA as noted in numerous reports from sub-Saharan Africa and other resource poor regions of the world is that, it is very expensive and often inefficient because it is time consuming [11, 12, 41]. According to Leitao and co-workers [42], the PCVA typically involves at least two physicians examining each record, with adjudication done by a consensus review or by a third physician. This may constitute a significant challenge in situations where there are numerous and often voluminous VA forms collected from a large population. Lozano and colleagues [12], noted that the usefulness of PCVA is based on the premise that physicians can interpret reported signs and symptoms correctly and occasionally household recall of Healthcare Experience (HCE) to accurately assign causes of death. This is not an easy venture because apart from the uncertainty associated with obtaining reliable household recall of HCE, the interpretation of the reported signs and symptoms highly depends on the skill and competence of the physician.

Available reports have indicated that operationalization differs by the number of physicians reading each VA, the methods used to adjudicate when different physicians disagree, and the procedures to map International Classification of Diseases (ICD) codes to the physician-assigned underlying cause of death [12, 43, 44]. Despite the fact that the use of PCVA is fraught with these drawbacks, a number of studies have shown that it still remains the only reliable method for determining COD from VA questionnaire data in locations where CRVS systems are lacking or dysfunctional [24, 31, 42]. Writing from experiences in the Nouna health and demographic surveillance site of Burkina Faso, Yé and colleagues [41] argued that the PCVA deficiencies should not prevent countries requiring information on causes of death from benefiting from the use of VA when no practical alternative for obtaining these data exists. However, Lozano and colleagues [12], advised that more rigorous standardization of questionnaire implementation, tests of diagnostic skill, and training might be able to improve concordance and perhaps increase Cause Specific Mortality Fraction (CSMF) accuracy.

### Validation of Physician-certified verbal autopsy with hospital records

As noted previously, the wider use of PCVA has raised a lot of concern regarding its validity. Consequently, a number of PCVA validation studies have been conducted in different LMICs to ascertain the extent of reliability of the method whereby the COD for individuals derived from PCVA is compared to the COD obtained from hospital records [19, 24, 25] (Table 1, Table 2 and Table 3). In the present VA validation review of child mortality in sub-Saharan Africa, a total of eight studies [31-33, 35, 37-40] out of the 14 selected studies focused on the comparison of PCVA with hospital records. Seven (87.5%) of the eight studies that compared the PCVA with hospital records were earlier studies conducted between 1990 and 2010. In Uganda, Mpimbaza and colleagues [31] compared PCVA with hospital records and noted that the sensitivity of PCVA in determining malaria death was 61% in Tororo and 50% in Kampala, its specificity was >88% in both locations, while the Positive Predictive Value (PPV) varied widely between the two locations; 83% and 34% respectively. In Ghana, PCVA performed poorly in stillbirth diagnosis and for neonatal deaths, its sensitivity was >60% for major causes of death while its specificity was 76% for birth asphyxia but >85% for prematurity and infection [32]. In Tanzania PCVA underestimated stillbirth and overestimated malaria death in post newborn [33]. Khan and colleagues [35] noted from their study in South Africa that PCVA had sensitivity, specificity and PPV of 69%, 96% and 90% respectively for communicable diseases whereas for non-communicable diseases the values were 75%, 91% and 86% respectively. The respective sensitivities and specificities for the five causes of death studied in Namibia by Mobley and co-workers were as follows: malnutrition 73% and 76%, cerebral malaria 72% and 85%, malaria 45% and 87%, diarrhea 89% and 61%, cough with dyspnea 72% and 64%, measles 71% and 85% [37]. In Kenya, PCVA detected the common cause of death with specificities >80%, but its sensitivity for measles, neonatal tetanus, malnutrition trauma-related deaths was >75%, while it was <50% for malaria, anemia, acute respiratory tract infection, gastroenteritis and meningitis.

From the forgoing, validation studies comparing PCVA to hospital records on child mortality in sub-Saharan Africa clearly demonstrate mixed and highly varied outcomes. This is consistent with results obtained from similar validity investigations conducted in other parts of the world in which the fraction of deaths where the true cause is accurately predicted varied from 0% to 95% for different causes [12]. Another major factor that compounds validation studies comparing PCVA to hospital records is that in most low-income settings the underlying quality of clinical diagnosis by physicians, hospital record keeping, laboratory and pathological services and medical imaging can be extremely poor [4, 11, 12]. Murray and colleagues have therefore described many of the validation studies as actually comparisons of two imperfect cause of death assignment approaches: low-quality hospital-assigned cause of death and the verbal autopsy [11]. Nevertheless, the validation of PCVA using hospital records should be viewed as a context-specific issue of which the accuracy and reliability depend on the level of healthcare the respondents have access to and the competence and knowledge of the physicians on the local disease aetiology and epidemiology [12, 29, 31]. Until newer, accurate, cheaper and more feasible methods of determining COD among children is developed, the sub-Saharan Africa will continue to rely on PCVA for establishing COD from VA questionnaire data, since most countries in the region are yet to make their CRVS very functional.

### Validation of physician-certified verbal autopsy with expert algorithm and InterVA

In recent years, efforts have been made to develop novel strategies for the interpretation of VA data that will serve as alternative to or replacement of PCVA for use in resource poor settings. Within the last decade a number of Computerized Coding of Verbal Autopsy (CCVA) methods for interpreting VA data have been investigated and validated and these include the algorithmic and InterVA methods [5, 14, 16, 21]. There are two types of algorithms, the expert-derived and the data-derived. According to Fottrell and Byass [45], expert algorithms consist of a set of predefined diagnostic criteria outlined by a panel of physicians, based on their experience or review of relevant literature, while data-driven algorithms are derived from existing data using standard statistical techniques. In the present review, a total of seven [16, 27-30, 34, 36] studies investigated the validation of VA by comparing either PCVA and algorithm or PCVA and InterVA (Table 1, Table 2 and Table 3).

In Nigeria, Adewemimo *et al.* [27] reported the outcome of the assessment of level of agreement between the Expert Algorithm Verbal Autopsy (EAVA) and PCVA and noted that both methods are very useful in providing direct estimates of the causes and determinants of neonatal and 1-59 month mortality. In Uganda, Mpimbaza and colleagues [28] compared the accuracy of PCVA, non-hierarchical and hierarchical algorithm in relation to the hospital diagnosis and observed high intra-rater reliability (83% agreement), the PCVA had high specificity for neonatal (>67%) and child death (>83%) while the hierarchical was better than the non-hierarchical algorithm. Comparing PCVA with expert algorithm in Niger, Kalter *et al.* [29], noted that cause distributions of neonatal deaths as determined by expert algorithms and the PCVA were similar, with the same top three causes by both methods and all but two other causes within one rank of each other. In Kenya, the COD was assessed by expert and data-derived algorithms and both were compared with hospital diagnosis, result showed that data-derived diagnostic algorithm gave comparable or better diagnostic accuracy than expert algorithm, but both gave high specificity [36]. These findings suggest that the algorithmic method performed well and could be described as an alternative to PCVA and its possible replacement. However, this outcome should be considered with some caution because a number of studies on expert opinion algorithm and data derived algorithm have shown conflicting levels of accuracy and sometimes even lower than that of PCVA [28, 31].

The use of the InterVA model to interpret VA data is increasingly gaining recognition and has been studied and validated in different resource scarce settings with encouraging outcomes [8, 16, 34]. In Kenya, PCVA and InterVA-4 were validated against hospital diagnosis of Sickle Cell Disease (SCD) among children aged 28 days to 14 years and result showed that 6% and 5% of deaths were attributed to SCD using PCVA and InterVA respectively [30]. Furthermore, agreement coefficient for SCD between PCVA and hospital diagnosis was 95.5% while that for InterVA was 96.9% [30]. In Ethiopia, the InterVA model was observed to be much less labour intensive than PCVA in determining COD among children and infants and offered 100% consistency [34]. The InterVA method is derived from Bayes' probability theorem and has been shown to be capable of achieving maximum consistency in interpreting VA data, requires relatively minimal time and labor resources, and freely available in the internet [16, 34, 46]. As with the algorithm method, InterVA has also been proposed as an alternative and possible replacement of the PCVA [15, 47-49]. However, Oti and Kyobutungi [16] argued that there is need to refine the InterVA model, continue its validation with more extensive data from different settings and give further thought to the interpretation and analysis of multiple causes of death for individual cases.

### Policy implication of VA validation

The need for sub-Saharan Africa to accelerate progress towards achieving the SDGs targets in child survival cannot be overstated. This is very important because if current trends continue, more than three quarters of all countries in sub-Saharan Africa will miss the under-five mortality target, and 13 countries in the region will not reach the target until after 2050 [1]. It is imperative therefore for the health systems on which child health interventions are deployed to be strengthened in the sub-region [50]. Accurate and reliable information on COD among children will continue to serve as one of the bedrocks for planning and health systems strengthening for improved child health outcomes in sub-Saharan Africa. As the countries in sub-Saharan Africa continues to battle with dysfunctional CRVS system, VA will remain the only viable option for the supply of child mortality data necessary for policy making [51].

Another important reason why VA will not only remain viable but also acceptable in sub-Saharan Africa is because of the myriad of concerns associated with incisional autopsy. Available reports from sub-Saharan Africa indicate that majority of the people will not normally give consent to performing incisional autopsy on their dead relatives. Some of the main reasons included: administrative challenges in requesting autopsy, delay in obtaining autopsy report, relatives of the deceased citing fear of mutilation, permission required from other family members, concerns about delaying the funeral, objection by the patient before death, and objection based on religious and cultural beliefs [52-55]. According to Oluwasola *et al.* [52], socio-demographic factors such as age, occupation, religion, ethnicity, and level of education significantly influenced willingness to give consent for autopsy. Because VA is generally the more acceptable autopsy in sub-Saharan Africa, the need for its standardization cannot be over emphasized.

The WHO is actively promoting the standardization of the VA tools to permit certification and coding commensurate with International Statistical Classification of Diseases and Related Health Problems, 10^th^Revision (ICD-10) and ascertain all causes of death with reasonable accuracy drawing on well-administered VA interviews [3]. Since WHO published the first three standard VA questionnaires in 2007, the tools have been revised and updated thrice (in 2012, 2014 and 2016) to improve cause-specific mortality data and to ensure consistency and comparability between countries [56]. However, because communities, healthcare planners, managers, researchers, global decision-makers and donors will continue to require VA data to inform burden of disease estimation and program evaluation, implies that COD estimates must meet high accuracy standards and be comparable over time and across countries [51].

## Conclusion

The outcome of this review clearly showed that none of the VA methods are a one-size-fits-all approach and like Leitao *et al.* [42] reported, none of the methods consistently outperformed the others across selected CODs, for both individuals and population-level COD assignment. There is a dire need to enhance the usefulness of VA data in facilitating evidence-informed policymaking and implementation process that will help to improve child health outcomes in sub-Saharan Africa. To achieve this, we subscribe to the suggestion of Thomas and colleagues [4] who noted that active collaboration of various stakeholders: governments, health authorities, communities and research groups, is highly required because this can foster engagement in, and coverage of, data collection and enhance the validity and utility of the process. This review was not without limitations. First, our use of only PubMed and the inability to use other databases for our search may have limited the number of potential publications for this review. However, we have confidence in the quality of publications from PubMed because it is a globally acclaimed data base for health research. Second, the variation in study design and reference standards employed in the various studies reviewed along with the differences in context of study areas could not permit adequate comparison across studies in order to make a generalized conclusion regarding the use of the VA methods to determine child mortality. More research is clearly needed to develop VA methods that are accurate and affordable for use in resource scarce setting like sub-Saharan Africa.

### What is known about this topic

- Despite the appreciable decline in global child mortality rate, sub-Saharan Africa remains the regions with one of the highest child mortality in the world;
- Reliable data on the Cause of Child Death (COD) is the cornerstone for evidence-informed health policy making towards improving child health outcomes;
- Verbal Autopsy (VA) has gained prominence as a strategy for obtaining COD information in most countries of sub-Saharan Africa because of absence of functional Civil Registration and Vital Statistics (CRVS) systems.

### What this study adds

- Physician-Certified Verbal Autopsy (PCVA) remains the most widely used VA method in sub-Saharan Africa;
- Validation studies comparing PCVA to hospital records, expert algorithm and InterVA demonstrated mixed and highly varied outcomes of which the accuracy and reliability are context-specific;
- None of the VA methods is a one-size-fits-all approach and none consistently outperformed the others across selected CoDs. More research is needed to develop VA methods that are accurate and affordable for use in sub-Saharan Africa.

## Competing interests

The authors declare no competing interests.
